# Evaluation of colour vision impairment during acute hypobaric hypoxia in aviation medicine: a randomized controlled trial

**DOI:** 10.1186/s12576-024-00898-4

**Published:** 2024-02-04

**Authors:** F. Liebold, W. Adler, S. Jansen, J. P. Klussmann, M. Meyer, L. Nehrlich, J. Schmitz, A. Vingerhoets, L. M. Heindl, J. Hinkelbein

**Affiliations:** 1 Department of Anaesthesiology und Intensive Care Medicine, University Hospital and Faculty of Medicine Cologne, Cologne, Germany; 2 Department of Ophthalmology, University Hospital and Faculty of Medicine Cologne, Cologne, Germany; 3 Department of Otorhinolaryngology, University Hospital and Faculty of Medicine Cologne, Cologne, Germany; 4 Department of Otorhinolaryngology, Head and Neck Surgery, University Hospital Essen, University of Duisburg-Essen, Duisburg, Germany; 5 Institute for Medical Informatics, Biometry and Epidemiology (IMBE), Friedrich-Alexander University Erlangen-Nuremberg, Erlangen, Germany; 6 Department of Medical and Clinical Psychology, Tilburg University, Tilburg, Netherlands; 7 Department of Sleep and Human Factor, German Aerospace Centre, Linder Höhe, 51147 Cologne, Germany; 8 Department of Anaesthesiology und Intensive Care Medicine, University Hospital and Faculty of Medicine Leipzig, Liebigstraße 20, 04103 Leipzig, Germany; 9 Johannes Wesling Klinikum Minden, University Hospital, Ruhr University Bochum, Bochum, Germany

**Keywords:** Colour vision, Pilot assessment, Hypobaric hypoxia, Aircraft, Eye

## Abstract

The digitization of aircraft cockpits places high demands on the colour vision of pilots. The present study investigates colour vision changes upon acute exposure to hypobaric hypoxia. The digital Waggoner Computerized Color Vision Test and the Waggoner D-15 were performed by 54 healthy volunteers in a decompression chamber. Respective altitude levels were sea level, 10,000 or 15,000 ft for exposure periods of 15 and 60 min, respectively. As for 60 min of exposure a significant decrease in colour perception was found between subjects at 15,000 ft as compared to the control group as well as between subjects at 15,000 ft as compared to subjects at 10,000 ft. No significant difference was found in the comparison within the 15,000 ft groups across time points pre-, peri-, and post-exposure. Thus, pilots appear to experience only minor colour vision impairment up to an exposure altitude of 15,000 ft over 60 min of exposure.

## Introduction

Prompt and accurate colour recognition is an essential component of pilot operations, and increasingly so as colour-coded electronic flight instrument systems (EFIS) becoming standard. General aviation pilots in the USA airspace are permitted to fly without the usage of supplemental oxygen at cabin pressures equivalent of up to 10,000 ft above sea level and up to 14,000 ft above sea level if the respective flight phase does not exceed the duration of 30 min. As for passenger safety purposes for cabin pressure altitudes above 15,000 ft enough oxygen must be available for all passengers for that part of the flight at those altitudes. Although the US Federal Aviation Administration (FAA) differentiates between different types of aircrafts, e.g., turbine engine powered airplanes or reciprocating engine powered airplanes, supplemental oxygen is generally required somewhere between 10,000 and 15,000 ft of cabin pressure equivalent [1–3]. In its “Literature review regarding the colour vision requirements for aircrew” the European Union Aviation Safety Agency (EASA) emphasized the challenges posed by the digital enhancement of cockpit systems. Thus, EFIS are generally said to be readable even for colour vision-deficient pilots, yet with potential confusion and delay in data processing. Integrated systems like the engine-indicating and crew-alerting system (EICAS) or the Electronic centralized aircraft monitor (ECAM) are explicitly using colour coding to support faster interpretation of the data provided [4]. Since most displays are self-illuminating, their visibility is no longer dependent on ambient lightning conditions. However, an increasing amount of information is shown on the panels at the same time, which can only be accomplished using an ever-growing number of hues and saturations [5]. In addition to the colour-coded information resources available inside the cockpit, warning and guidance systems outside the aircraft may also cause errors in interpretation. These include airfield lighting, e.g. taxiway signs, navigation light, e.g. position lights, and Visual Approach Slope Indicator (VASI), e.g. Precision Approach Path Indicator (PAPI) [6, 7]. Chromatic sensitivity of the central nervous system has been reported to deteriorate in both simulated and non-simulated hypoxic conditions. Referring to widespread diseases like glaucoma or diabetic retinopathy scientific efforts was primarily made regarding long-term effects of hypoxia in the visual system. Several pathophysiological mechanisms were considered responsible for the deterioration of visual acuity and colour vision under hypoxic conditions which may also be crucial in the context of acute exposure to hypoxia.

Thereby, the emphasis is generally on the retina. The retina is the most oxygen-consuming tissue in the human body per unit weight. High-altitude-related changes of the retina are commonly known as high-altitude retinopathy. This may include, e.g.

- increasingly dilated retinal vessels with increased permeability due to an upregulated expression of endothelial nitric oxide synthase (eNOS) and vascular endothelial growth factor (VEGF) and subsequent haemorrhaging in the inner retinal and pre-retinal space [8, 9],
- optic disc swelling due to NO-induced increase in the cerebral blood flow and decreased blood–brain barrier integrity as a consequence of hypoxia induced Na^+^/K^+^ ATPase failure and free radical formation damaging vessel basement membranes [8, 10],
- optic disc hyperaemia [11],
- haemorrhages in the vitreous cavity [11].

However, whilst correlations between these pathophysiological changes and clinical symptoms could already be postulated—e.g. between optic disc swelling and the Acute Mountain Sickness Score (AMS) [12]—concrete coherencies to colour vision deterioration have not yet been proven. It may nevertheless be added that the areas of the greatest retinal oxygen consumption are the inner segments (neuroepithelial layer of the retina) as well as the inner and outer plexiform layers. It is here where the photoreceptors and the region of synapse formation between the photoreceptors and the second and third neurons of the visual pathway can be found. Located in the outer retinal layer these structures are supplied with oxygen via the capillaries of the choroidal circulation. Unlike with the inner retinal layer, where oxygen is supplied directly via the central retinal artery, oxygen must diffuse over a greater distance to the target region, which requires a certain minimum concentration [13–16]. Besides, morphological differences between the distinct cone types were postulated as a possible influential factor for the differential hypoxia sensitivity of single colour channels. S-cones represent only 5–6% of all cones and are predominantly located around the fovea centralis and therefore supplied with blood by the choroid [17, 18]. The elevated vulnerability to hypoxia of this cone type could already be demonstrated in several experiments showing distinct changes in action potentials in the electroretinography as well as deteriorated colour vision in the blue range (the absorption maximum of S-cones ranges between 440 and 450 nm) [19–22]. Some authors also suspect an increased metabolic turnover of S-cones predisposing to hypoxia intolerance [23]. To date there is very little known about the energy metabolism of the post-retinal optic tract from the retina to the visual cortex. It is considered a certainty that the latter is one of the most energy consuming area of the brain, whereas the bulk of ATP is required for sustainment of Na^+/^K^+^-ATPases to repolarize membranes after depolarization. The lateral geniculate nucleus (LGN) is the main relay station between the retina and the primary visual cortex and is primarily supplied by the thalamogeniculate arteries. LGN neurons are very sensitive to the level of retinal input whilst at the same time energy metabolism and neuronal activity are tightly affiliated. The primary visual cortex is the first cortical area involved in colour processing. It offers a high density of penetrating blood vessels. In fact, layer IV of this area impresses with the highest level of cytochrome c oxidase found in humans. Here, as well in other layers of the cortex 2-deoxyglucose uptake positron emission tomography and functional magnetic resonance imaging could show a positive correlation amongst blood flow, glucose utilization, capillary density, cytochrome-c-oxidase levels and neuronal activation. Beyond the primary visual cortex, colour vision processing continues in extrastriate visual cortical areas with extensive interconnections. The architecture of the so-called colour centre involving the primary visual cortex as well as higher order visual processing (primarily located in the occipital lobe) is complex and still under investigation [24].

There is clear evidence that especially long-term exposure to hypoxia as well as mesopic conditions lead to a functional impairment of retinal photoreceptors. Based on the colour vision tests applied, the duration of the (simulated) altitude exposure and illumination conditions, thresholds values could be determined above which a functional limitation of colour detection and discrimination can be assumed. As per available data this is between 8000 and 10,000 ft [19, 25–27]. However, there is very little known about the effects of moderate hypobaric hypoxia in photopic conditions for short time exposure, as needed for application to aviation safety. In the context of this paper, the term “moderate hypoxia” refers to exposure of a normal healthy adult to the aircraft cabin or pressure chamber environment during routine commercial air travel or simulated flights not exceeding 15,000 ft of ambient pressure. Both private and professional pilots are required to undergo colour vision testing in advance of their initial certification as well as periodically in the course of their flight activity. Due to the general wording for colour vision standards of the International Civil Aviation Organization (ICAO) respective requirements for certification and the applied colour vision tests differ significantly between countries or certification authorities [28, 29]. Colour vision tests can be classified in different ways according to their differential-diagnostic abilities or the way colours are presented to the observer. Screening tests like the Ishihara pseudoisochromatic plate test detect colour vision deficiencies without providing information about the extent of the defect or the specific colour range that is affected. They feature a high sensitivity but low specificity. In contrast, confirmation tests (precision tests) like the Nagel Anomaloscope allow detailed characterization about a colour anomaly. Test systems for the detection of dyschromatopsia can also be classified into two major groups:

- Tests with pigment colours (e.g. pseudoisochromatic plates or panel tests)
- Spectrum tests with coloured lights, (e.g. Nagel Anomaloscopes, partly Lantern tests)

In addition, these approaches can be subdivided into qualitative (e.g., pseudoisochromatic plates) or quantitative (e.g., Nagel Anomaloscope) [5] as well as into analogue and digital tests. The most commonly used test systems are analogue pseudoisochromatic plates. The FAA accepts, e.g. Dvorine plates (second edition, 15 plates), Ishihara plates (14-plkate edition, 24-plate edition, 38-plate edition) and Richmond pseudoisochromatic plates (1982 edition). Lantern tests are also commonly used. Hereby, the Farnsworth Lantern test can be found amongst the acceptable test systems by the FAA [30]. The majority of the test methods is cost intensive and time consuming [31]. On the one hand, this is due to the expensive test systems themselves, which, as medical products, have to comply with high requirements in terms of product quality and standardization. On the other hand, analogue test systems must be performed under standardized light conditions, which require special light booths that may in turn cost several thousand Euros. Digital colour vision tests such as the Waggoner Computerized Color Vision Test (CCVT) and the Waggoner D-15 (WC-D15) (Waggoner Color Vision Testing, LLC © (Rogers, USA) offer an alternative in the future, since they are less demanding in terms of environmental conditions and at the same time can be used in part on commercially available display devices [7, 32]. However, such products are still a relatively new development and have not yet been tested under many conditions crucial for scientific purposes.

The aim of the present study was primarily to investigate colour vision changes amongst healthy volunteers at moderate hypoxia in a decompression chamber. Unlike with existing data an aviatic approach applying rapid ambient pressure build-up and release (simulating take-off and landing of an aircraft) and short-time overall exposure was implemented. It was hypothesized that exposure to acute hypobaric hypoxia will significantly reduce colour vision ability and further that an increase in exposure time will amplify the respective effect.

## Material and methods

The present study complied with the Declaration of Helsinki and was approved by the ethical committee of the University of Cologne, Germany (No. 18-045).

### Inclusion and exclusion criteria

*N* = 54 subjects were randomly assigned into five different groups, determined by time of exposure, simulated altitude and ocularity (mono- or binocular vision):

- ground control binocular (*n* = 12, Bi/GC)
- ground control monocular (*n* = 11, Mo/GC)
- 10,000 ft, 60 min, monocular (*n* = 10, Mo/60/10,000)
- 15,000 ft, 15 min, binocular (*n* = 11, Bi/15/15,000)
- 15,000 ft, 60 min, monocular (*n* = 10, Mo/60/15,000)

**Fig. 1:**
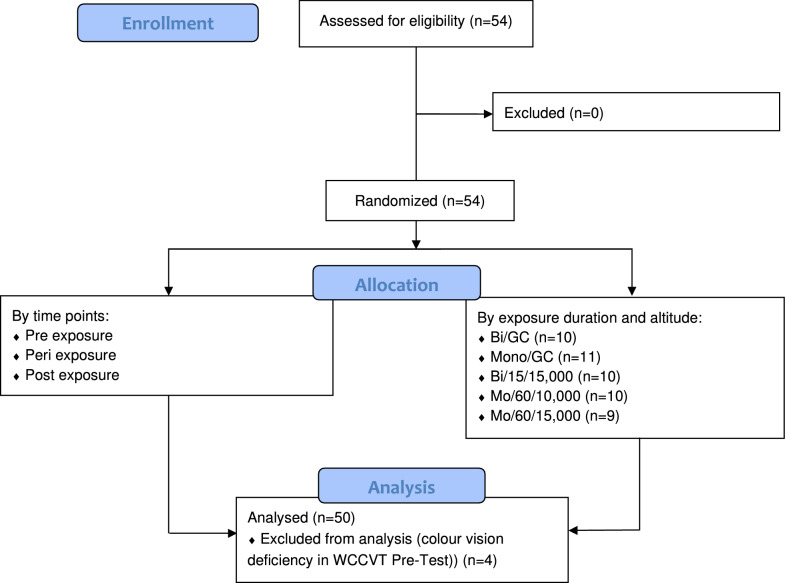
Consort flow diagram—study design

Mean age of the subjects was 29, 63% were male and 37% were female. Subjects were assigned to exposure groups in a randomized approach and were screened for acute or chronic disease before being assigned to one of the study groups. Informed consent was obtained from all subjects involved in the study. For the participation in the study subjects had to be at least 18-year old, having sufficient language competence in German or English and needed to be able to read, understand and sign the consent form. Participants who suffered from cardiovascular, pulmonary, neurological, or ear, nose, and throat disease were excluded from this study from the outset. Also, individuals who were pregnant or suffering from cold symptoms did not meet the inclusion criteria and were, therefore, excluded (Fig. 1).

### Study setting

**Fig. 2:**
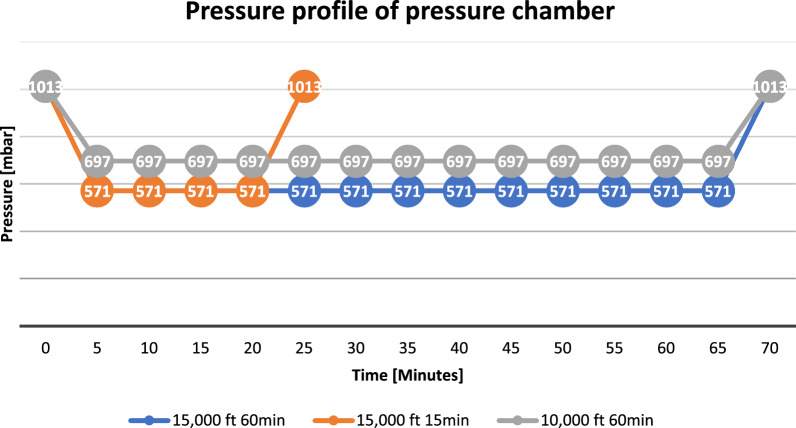
Pressure chamber profile: ambient pressure conditions of the three different intervention groups over time, blue: 15,000 ft, 60 min, monocular; orange: 15,000 ft, 15 min, binocular; grey: 10,000 ft, 15 min, monocular

Glasses were allowed at all times to ensure optimal visual acuity. In the monocular conditions the eye was occluded with an ophthalmic patch. The experimental set-up was divided into three phases with respect to hypoxia exposure: pre-, peri-, and post-phase. To detect possible learning effects due to repeated testing cycles that could possibly mask colour vision deterioration due to hypoxia the GC groups were implemented where colour vision tests were performed three times in a row with the altitude chamber inactive. As for the exposure groups colour vision tests were conducted before exposure (Pre, no hypoxia), during exposure (Peri, induced hypoxia at respective altitude level) and after exposure (Post, no hypoxia). All phases were passed through directly one upon the other to ensure adequate simulation of flight stages. Exposure groups had a peri-phase lasting exactly 15 and 60 min, respectively. All subjects were monitored via a pulse oximeter (pulox-Po-100-solo, © Novidion GmbH, Cologne, Germany) on the fingertip of one index finger at all times. The compression chamber (HAUX, Karlsbad, Germany, accessible for one person and operated by automatic pressure profile) was located at an altitude of 3.3 ft above sea level (1006.9 hPa). The simulated altitudes of 10,000 ft and 15,000 ft were equivalent to atmospheric pressures of 696.9 hPa and 571.87 hPa, respectively. Simulated ascent and descent lasted 5 min to each of the three exposure groups. Hence, pressure alternation was 85.6 mbar/min and 63.3 hPa/min, respectively (Fig. 2).

### Colour testing

The combination of a pseudoisochromatic plate test and an arrangement test was considered the most appropriate testing method to evaluate the study objective. Pseudoisochromatic plate tests such as Ishihara Plates are generally used for screening of colour vision defects in aviation and are based on the principle of colour confusion and colour saturation. They are fast and intuitive in use which was crucial especially for the 15-min hypoxia cohort. The fact that precise characteristics of a colour vision defect cannot be provided or quantified by the test was secondary to the present investigation as the aim of the study was to detect any kind of colour vision deterioration. A pseudoisochromatic plate test can be followed by an arrangement test such as the Farnsworth D15 to further evaluate the severity of a colour deficiency. The Farnsworth D15 was developed to distinguish between moderate and severe colour deficiency and therefore dichotomises subjects into two groups, “pass” and “fail” [33]. The test is based on the ability to isolate and arrange differences in various colour targets with constant value and chroma that cover all the visual hues included in the Munsell colour system [5].

All subjects underwent colour vision testing using the Waggoner Computerized Color Vision Test (WCCVT) and the Waggoner D15 (WC-D15). The WCCVT is a redesigned pseudoisochromatic plate test with a screening section designed to detect protan, deutan, and tritan colour vision defects (CVDs). Subjects were first presented with 25 cards for the purpose of screening, on which they had to correctly identify numbers against a coloured background (similar to Ishihara plates). This was followed by a section with 12 plates for explicit testing of the tritan axis (as specified and not alterable by the software). The number of correctly recognized plates could thus be used for statistical analysis.

The WC-D15 comes as a digital version of the conventional Farnsworth–Munsell Dichotomous D-15 and provides the respective Moment of Inertia according to the quantitative scoring technique (Colour Difference Vector Analysis) by Vingrys and King-Smith [32, 34]. The authors used the 1976 CIELUV colour space of Wyszecki and Stiles for interpretation of the results. Accordingly, the method calculates colour difference vectors between adjacent caps to provide information about the specific nature of a defect [35]. There are three parameters fully describing the subjects score on the panel test. The Confusion Index (C-index) defines the severity of the error with 1 indicating perfect arrangement. A cap that is incorrectly placed on the opposite side of the HUE circle is causing a crossing within the circular aggregate. The Scatter Index (S-index) is a measure of how regularly crossings are aligned. Ultimately, the Angle of Inertia indicates the type of defect with protan angles larger than 0 and deutan angles smaller than 0 [32, 34].

**Fig. 3:**
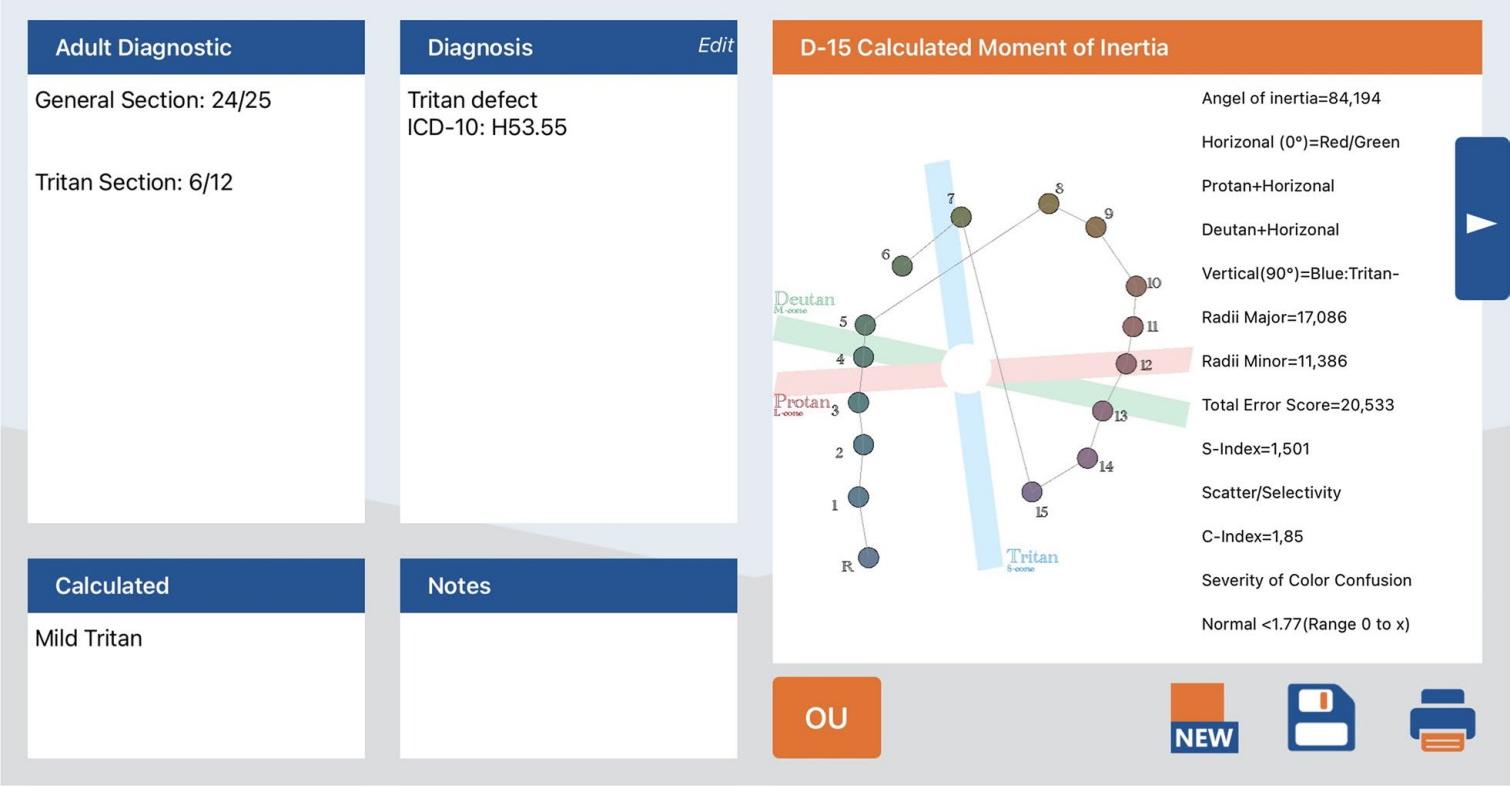
WCCVT and WC-D15 analysis of a tritan defect example subject. Left: result sheet of the WCCVT showing the number of correctly identified plates in the sections General and Tritan; right: result sheet of the WC-D15 graphically showing the cap transpositions on the tritan colour confusion axis including the corresponding calculated error parameters [from Waggoner Color Vision Testing software, LLC © (Rogers, USA)]

In addition, a Total Error Score (TES) can be obtained indicating the average colour difference between caps (Fig. 3).

As for the monocular groups, both eyes were tested in succession by the software. Results were later analysed individually and if not differentiated significantly, averaged for statistical analysis. Subjects who were identified as colour vision deficient during the Pre-testing phase were excluded from the analysis. Both tests were third party validated and are amongst current tools recommended by FAA and EASA for aircrew colour vision assessment [5, 32, 36, 37].

The Waggoner software was run on an iPad Air 2019 (10.5-in.) device (Apple©, Cupertino, USA) with the IOS 12.3 operating system and the recommended settings (True Tone deactivated, display brightness, eye-display distance 60–76 cm) applied. The illuminance level inside the altitude chamber was 4 lx to avoid glare and reflections on the display.

### Statistical analysis

Statistical Analysis and data collection were performed using SPSS (IBM® Armonk, New York, USA). Based on the results of previous studies, we have calculated a sample size of *n* = 10 participants per group. *T* test was used with an alpha error of 5% and a beta error of 80%. Mann–Whitney *U* test was applied for comparison between two groups and the Friedman test for comparison between time points (Pre, Peri, Post). In case of significant results by Friedman testing, the comparison of the corresponding pairs was done by Wilcoxon signed-rank test (Table 1). The Waggoner Software, if set to monocular mode, always starts testing with the left eye. Thus, comparison between eye sides could be conducted to detect possible learning effects using paired *t* test. Linear regression analysis was further calculated to detect a possible correlation between test results and SpO_2_ saturation levels as for the exposure groups during the time point Peri.

**Table 1:** Study comparisons and corresponding statistical analysis: statistical tests that were applied for the statistical analysis of each comparison based on recommended allocation requirements

Outcome	Comparison	Data type	Statistical model
Colour vision ability—WCCVT	Comparison between two study groups for one particular time point, i.e. Mo/60/10,000 peri vs. Mo/60/15,000 peri	Number of correctly identified plates	Mann–Whitney *U* test
	Comparison between particular time points within a study group, i.e. Mo/60/10,000 pre vs. Mo/60/10,000 peri		Friedman test
	Comparison between eye side within a particular study group, i.e. Mo/60/10,000 pre left eye vs. Mo/60/10,000 pre right eye		*t* test
	Correlation between scoring levels of the time point Peri and SpO_2_ saturation levels	Number of correctly identified plates and SpO_2_ saturation levels	Linear regression
Colour vision ability—WC-D15	Comparison between two study groups for one particular time point, i.e. Mo/60/10,000 pre vs. Mo/60/15,000 pre	S-Index, C-Index	Mann–Whitney *U* test
	Comparison between particular time points within a study group, i.e. Mo/60/10,000 pre vs. Mo/60/10,000 peri		Friedman test
	Comparison between eye side within a particular study group, i.e. Mo/60/10,000 pre left eye vs. Mo/60/10,000 pre right eye		*t* test
	Correlation between scoring levels of the time point Peri and SpO_2_ saturation levels	C-Index	Linear regression

A result was considered significant with *p* value less or equal 0.05.

### Complex directional hypotheses

The independent variables “simulated altitude”, “exposure time” and “eye side” were altered to measure the effect on the dependent variables “WCCVT test results” and “WC-D15 test results”. Research hypotheses:

- Exposure to a hypobaric hypoxia equivalent to 10,000 ft or 15,000 ft respectively will significantly reduce colour vision in subjects based on the principle of colour confusion and colour saturation (as measured through WCCVT) and the ability to isolate and arrange differences in various colour targets (as measured through WC-D15).
- An increase in exposure time to hypobaric hypoxia will significantly amplify colour deterioration in subjects based on the principle of colour confusion and colour saturation (as measured through WCCVT) and the ability to isolate and arrange differences in various colour targets (as measured through WC-D15).

The null hypothesis states that there is no statistical difference between groups based on the stated research hypotheses.

## Results

Four subjects were excluded from the study as they showed colour vision deficiencies in the WCCVT Pre-testing. Correspondingly, 240 colour vision tests on 50 subjects were conducted (Fig. 1). Considering monocular cohorts, results could be averaged between eye side throughout as paired *t* tests showed no significant differences for any of the respective comparisons.

Subjects of Bi/GC obtained an average of 24.4 plates of the General section and 12 plates of the tritan section in the WCCVT. The average C-index was 1.02. Only two subjects failed to achieve accuracy in three consecutive tests performing the WC-D15. Therein, cap transpositions were entirely found on the tritan axis (67.6° < angle of inertia < 76°) with a mean S-index of 1.59 and low TES (12.3 < TES < 13.9). The mean sO_2_ was 98%, with two subjects showing faulty measurements that therefore could not be recorded.

Subjects of Bi/15/15,000 correctly identified an average of 24.4 (Pre), 24.6 (Peri), and 24.8 plates performing the WCCVT General. As for the WCCVT tritan section and the WC-D15, all subjects obtained accuracy. Comparing Bi/GC vs. Bi/15/15,000, no significant difference could be shown for any of the three tests applied over three phases of a simulated flight. The mean sO_2_ peri was 75.4%.

Subjects of Mono/GC obtained an average of 24.3 and 11.8 plates in the WCCVT General and tritan section, respectively. The mean C-Index was 1. The mean sO_2_ peri was 97.7%. Comparison between the two GC groups showed no significant differences regardless of test methods.

As of the Mo/60/10,000 cohort, mean WCCVT General were 24.3 (Pre), 24.3 (Peri), and 24.4 (Post) and mean WCCVT tritan were 11.8 (Pre), 11.9 (Peri), and 11.8 (Post). Mean C-Indexes were 1.02 (Pre), 1.01 (Peri), and 1 (Post) with only one subject not achieving accuracy. Therein, cap transpositions were made on tritan and deutan axes (57.8° < angle of inertia < 67.4°) with mean S-Index of 1.62 and low TES (12.4 < TES < 13.8). The mean sO2 peri was 87.6%. No significant difference could be found comparing Mo/60/10,000 vs. Mo/GC in respect of test method and phase of exposure.

**Fig. 4:**
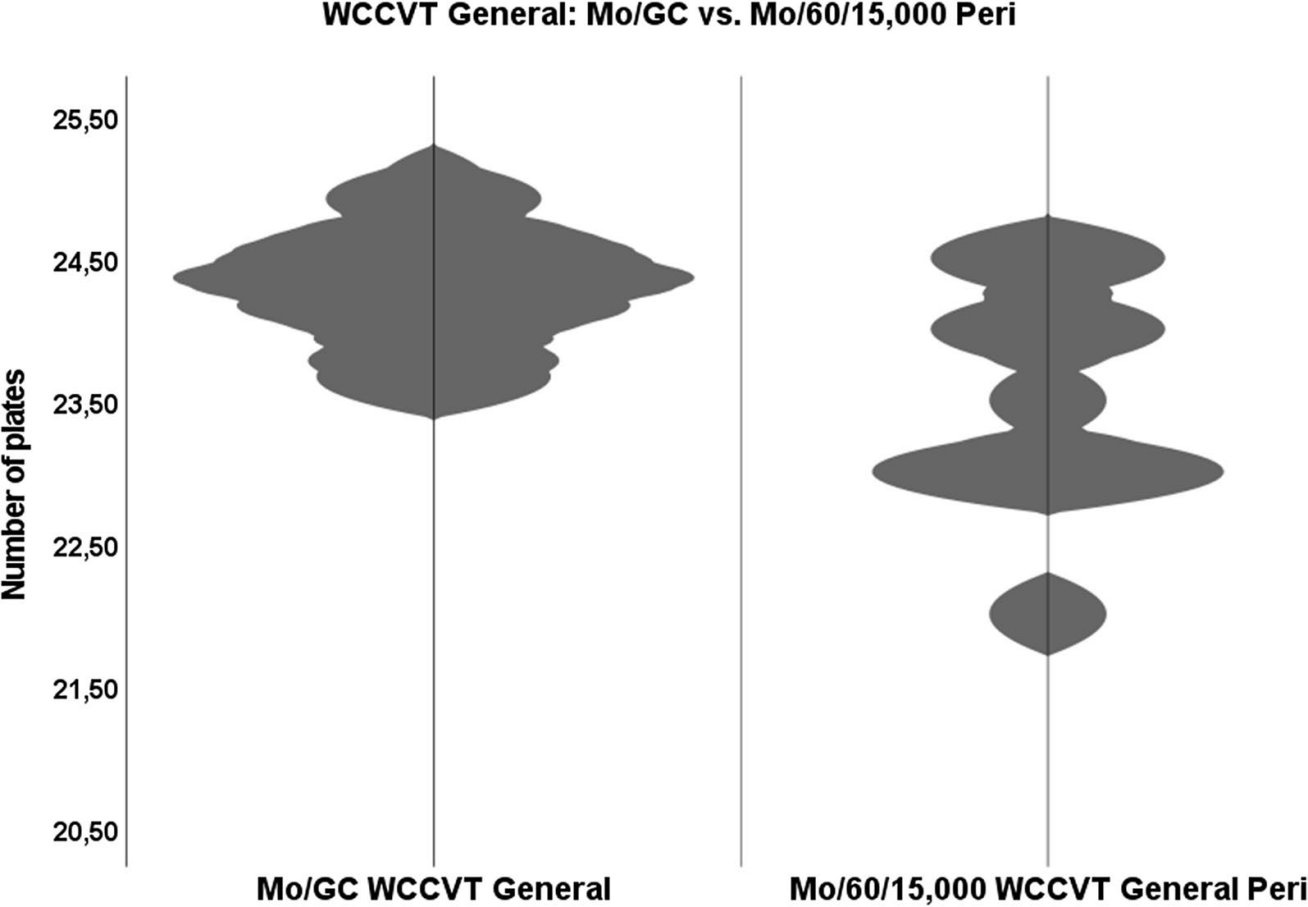
WCCVT general—comparison between Mo/GC and Mo/60/15,000 at time point Peri: violin plot showing the central tendency between Mo/GC WCCVT general and Mo/60/15,000 WCCVT General Peri. The difference was significant

The Mo/60/15,000 cohort did perform inferiorly to some extent. Mean WCCVT General were 23.9 (Pre), 23.5 (Peri), and 24.1 (Post) and mean WCCVT tritan were 11.67 (Pre), 12 (Peri), and 11.78 (Post). Mean C-Indexes were 1.1 (Pre), 1.07 (Peri), and 1.01 (Post) with three subjects not achieving accuracy. Within which, caps transpositions were made arbitrarily across time points on the tritan and deutan axes (-75.9° < angle of inertia < 79°) with mean S-index of 1.61 and moderate TES (12.4 < TES < 20). The mean sO_2_ peri was 74.4%. Mann–Whitney *U* testing showed a significant difference (*p* = 0.025) between Mo/60/15,000 and Mo/GC in the WCCVT General section as of exposure phase Peri (Fig. 4).

**Fig. 5:**
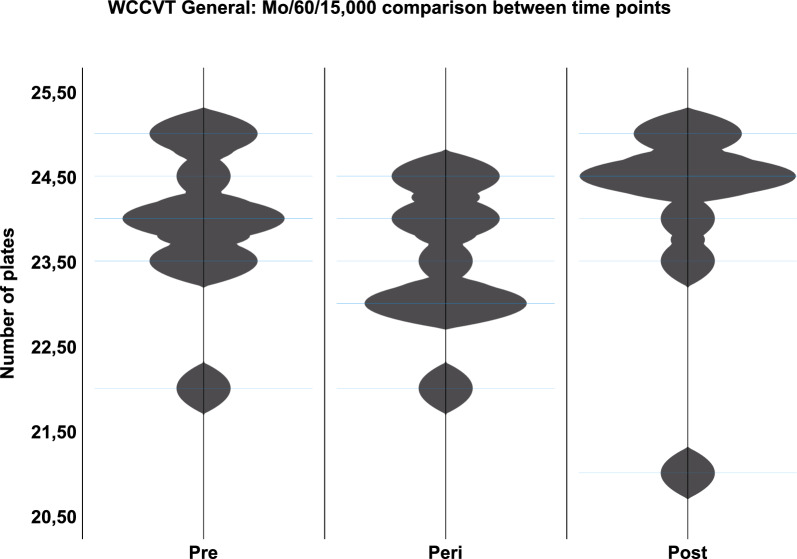
WCCVT general—comparison between time points of Mo/60/15,000: violin plot showing the central tendencies between time points within Mo/60/15,000 WCCVT general. The differences were not significant

Subsequently, time points within cohorts were compared with one-another. Again, no significant differences were found in any of the groups. However, the greatest central tendencies between time points were found in Mo/60/15,000 (Fig. 5).

**Fig. 6:**
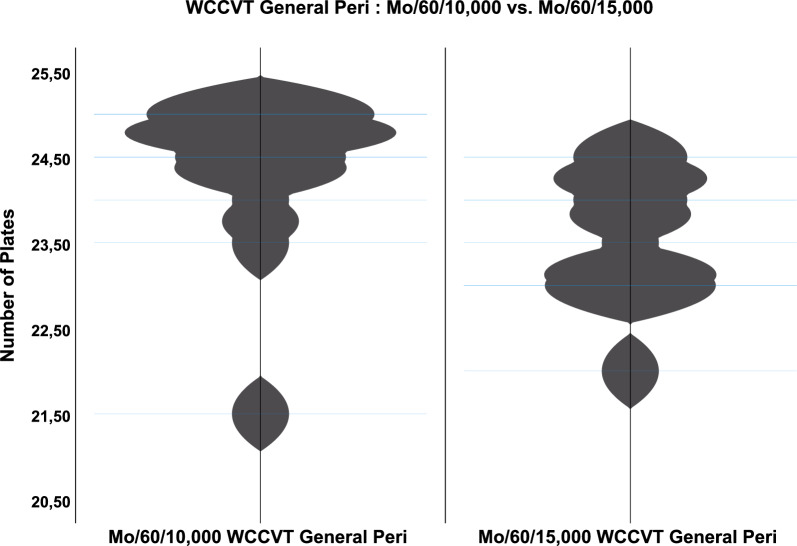
WCCVT General Peri—Mo/60/10,000 vs. Mo/60/15,000: violin plot showing the central tendency between Mo/60/10,000 WCCVT General and Mo/60/15,000 WCCVT General Peri. The difference was significant

At last, the two monocular exposure groups were compared to investigate a possible effect of altitude at equal times of exposure. Herein, a significant difference was found between the groups in the WCCVT General at time point Peri (*p* = 0.035, Fig. 6).

No significant difference could be found comparing both eyes amongst time point Pre in the exposure groups and between the three time points in the control groups, respectively. The same applied for Bi/GC between all three time points (Table 2). Linear regression analysis could not detect any correlation between test results and SpO_2_ saturation amongst subjects in the exposure groups (Table 3).

**Table 2:** Colour vision test results

Exposure group	Mean values of WCCVT (standard deviation, SD)		Values of WC-D15 (standard deviation, SD)		Mean O_2_ values (standard deviation)
	General	Tritan	C-Index	TES	
Bi/GC	24.4 (0.93)	12 (0)	1.02 (0.06)	12.3 < TES < 13.9 (0.43)	98.1 (0.83)
Mo/GC	24.3 (0.80)	11.8 (0.58)	1 (0)	11.416 (0)	97.7 (1.02)
Bi/15/15,000					
Pre	24.4 (0.096)	12 (0)	1 (0)	11.416 (0)	97.9 (1.2)
Peri	24.6 (0.70)	12 (0)	1 (0)	11.416 (0)	75.5 (4.07)
Post	24.8 (0.44)	12 (0)	1 (0)	11.416 (0)	98.2 (0.63)
Mo/60/10,000					
Pre	24.3 (1.21)	11.8 (0.44)	1.02 (0.07)	11.416 < TES < 13.82 (0.57)	97.8 (0.42)
Peri	24.3 (1.21)	11.9 (0.37)	1.01 (0.03)	11.416 < TES < 12.45 (0.23)	87.2 (2.7)
Post	24.4 (0.88)	11.8 (0.44)	1 (0)	11.416 (0)	98.1 (0.88)
Mo/60/15,000					
Pre	23.9 (1.06)	11.65 (0.49)	1.10 (0.22)	11.416 < TES < 20.045 (2.12)	97.67 (1)
Peri	23.5 (1.34)	12 (0)	1.07 (0.15)	11.416 < TES < 16.125 (1.27)	75.44 (3.47)
Post	24.1 (1.47)	11.78 (0.43)	1.02 (0.07)	11.416 < TES < 12.39 (0.31)	98.22 (0.67)

**Table 3:** Test score correlation with SpO_2_ saturation levels: correlation between scoring levels in the WCCVT and WC-D15 of the time point Peri and SpO_2_ saturation levels using a linear regression model and analysis of variance (ANOVA)

Exposure group	SpO_2_—test scores’ correlation	
	*R*^2^	ANOVA significance level
Mo/60/15,000 Peri		
WCCVT general	0.017	0.74
WCCVT tritan	Accuracy	Accuracy
WC-D15 C-index	0.065	0.51
Bi/15/15,000 Peri		
WCCVT general	0.108	0.35
WCCVT tritan	0	0.97
WC-D15 C-index	Accuracy	Accuracy
Mo/60/10,000 Peri		
WCCVT general	0.036	0.6
WCCVT tritan	0.021	0.69
WC-D15 C-index	0.011	0.78

None of the two null hypotheses could be rejected.

## Discussion

The present study is the first analysis of colour vision changes under short-term altitude exposure using digital colour vision assessments on a tablet computer. It contributes to the pre-existing literature by adapting test settings specifically to the requirements in aviation and therefore applying simulated flight profiles and digital test methodologies referring to EFIS. The study indicated no change in colour vision according to the WCCVT und the WC-D15 over an exposure period of up to 60 min at a simulated altitude of up to 10,000 ft. As for a simulated altitude of 15,000 ft. Over 60 min, there was evidence for deterioration of colour vision. However, results were not significant in the crucial comparison across time points within the respective exposure group.

### Ocularity

The comparison between the two GC (monocular vs. binocular) groups showed no significant correlation. There is inconsistent evidence to date that colour vision differs between eye sides in healthy subjects. Mäntyjärvi administered the Farnsworth–Munsell 100-Hue (FM-100 Hue) on 160 subjects and found no significant differences between right and left eye. The authors therefore concluded that for vocational purposes binocular testing may be sufficient [38]. Costa et al. indicated the absence of binocular summation of colour discrimination using the Cambridge Colour Test (CCT) [39]. However, several other studies came to contrary results. Verriest et al. postulated worse performance of 116 subjects conducting the FM-100 Hue monocularly compared to binocularly [40]. Jiménez et al. found lower contrast thresholds in binocular subjects for chromatic red-green or blue-yellow gratings [41]. Conolly et al. reported lower thresholds in binocular than in monocular viewing conducting the Colour Assessment and Diagnosis (CAD) test on 11 subjects [23]. Irrespectively monocular testing offers some advantages in a scientific context. Acquired colour vision deficiencies can occur unilaterally and colour deficient subjects can, therefore, be missed when testing binocularly. In addition, testing each eye at a time increases the total number of tests applied and consequently reduces possible bias such as misapplication upon first-time conduction. Conversely, an equally possible learning effect when running a test multiple times in a row can be detected. In the present study no learning effect was observed with repeated use of the assessments. Moreover, Verriest et al. suggested applying monocular testing on the FM-100 Hue using the second eye of the same observer as a control. The subject then serves as his or her own control which reduces bias due to interindividual variability [40]. Ultimately, pilots with monocular vision are to be considered for medical certification of any class according to the FAA. At the time being, there is no evidence whether monocular vision predisposes to deteriorated colour perception upon exposure to high altitude as seems to be the case with mesopic conditions [42].

### Hypoxia and reduction in ambient pressure

The comparison between the two GC and Bi/15/15,000 or Mo/60/10,000 showed no significant differences across test methods and time points. This is in contrary to a study by Karakucuket et al. on 16 high-school students conducting the FM-100 Hue. The group found a significant increase in error numbers in the blue–yellow axis at 9840 ft. However, ascent time was 3 h compared to 5 min in the present study and exposure duration was considerably extended even compared to Mo/60/10,000 [26]. Application of the results on non-pressurized aircrafts is therefore limited. Vingrys et al. reported a statistically significant decrease in colour discrimination on the FM-100-Hue and the P-N anomaloscope of two subjects at a simulated altitude of 12,000 ft [43]. However, in this study the altitude adaption period was 15 min at minimum (compared to five to ten minutes in the Bi/15/15,000 group) and the whole experiment lasted 3 h with breaks between tests. The difference in acclimatization time of about 10 min and more can be considered as a possible explanation for the conflicting results of the two investigations. As for Mo/60/10,000, discrepancies between the two studies could further be explained by the differences in altitude.

Subjects of Mo/60/15,000 showed a deterioration in colour vision with significant difference compared to the respective GC and non-significant differences between time points. This finding is in concordance with previous studies, although no study has yet been found in which colour vision tests were conducted at the exact altitude of 15,000 ft [26, 27, 43, 44].

### Type of colour vision test

Whilst there is agreement that moderate hypoxia decreases colour vision, there is wide variation in the assessment of the extent of this influence. Hovis et al. found only a minor influence of hypoxia on chromatic discrimination averaged across observers with normal colour vision and trichromats with red-green defects at a simulated altitude of 12,400 ft and an exposure duration of up to 4 h using the Colour Assessment and Diagnosis (CAD). The Cambridge Color Test (CCT) and Cambridge Color Vision Test (CCV) showed no significant changes. SpO_2_ values showed a great interobserver variability and varied from 79 to 95% [45]. Wawolumaya reported a significant change in the ability of correctly recognizing Ishihara Plates of 49 pilots of the Indonesian Air Force at a simulated above moderate altitude of 18,000 ft. The simulated flight profile of their study was presumably comparable to the present study. However, even under hypoxic conditions the colour vision ability of their subjects was still in a normal range. Mean SpO_2_ value was 68.2% at 18,000 ft and therefore below the mean SpO_2_ of 74.44% in the Mo/60/15,000 Peri cohort of the present study [44].

In general, it is important to note that the results of colour vision testing seem to be partially dependent on the type of colour vision test applied. The Waggoner CCVT is a pseudoisochromatic panel test whilst the WC-D15 comes as a colour discrimination arrangement test. The former type of test has already been critically evaluated in several studies with reference to altitudinal research. Vingrys et al. conducted three test methods simultaneously on two healthy subjects in a decompression chamber at simulated 12,000 ft., therefore Hardy–Rand–Rittler pseudoisochromatic plates (HHR), the FM-100-Hue and the P-N anomaloscope. No loss of colour vision could be detected by the HHR, while both alternative test methods showed clear abnormalities [43]. The authors also referred to a study by Wilmer and Berens, who were also unable to detect any loss of colour vision using Stillings Plates, even though a simulated altitude of 20,000 ft was applied to 5 subjects [46]. Hence, the assumption was made that pseudoisochromatic plates might not be sensitive enough to detect subtle changes as found for moderate altitude exposure. The present study can be considered as an indication that this could also apply to the Waggoner WCCVT. As for the Farnsworth D-15, literature data are also very inconsistent. Leid et al. examined mountaineers at altitudes of up to 23,000 ft using the Farnsworth desaturated D-15 test and found no significant abnormalities. This is all the more surprising as the two subjects of the 23,000 ft group suffered from acute altitude sickness and the exposure duration was significantly increased compared to the present study. Unfortunately no data on SpO_2_ were provided by the authors [47]. Marechal et al. in their comparison of Pilots’ Medical Assessments stated that the D-15 has only limited suitability for aeromedical use. In their study, subjects with colour vision deficiencies could only be insufficiently detected compared to other test methods (e.g. Nagel anomaloscope, CAD, Lanthony 15 Hue) and flight suitability could have been falsely implied [48]. The FM-100-Hue, as used in many of the studies mentioned above, is a widely used colour vision test suitable for the detection of both congenital and acquired colour vision changes [49]. In view of the large number of studies that have detected colour vision changes upon altitude exposure using the FM-100-Hue, a sensitivity can be assumed for this field of application. However, Bassi et al. compared the FM-100-Hue with the Farnsworth D-15 and L’Anthony D-15 desaturated using logistic regression. The groups concluded that the two D-15 assessments could adequately determine the severity of the colour deficits although correlations of TES, S-Index and C-Index did not reach 100% [35]. Nonetheless, given this background, it is not surprising that no significant changes could be detected by the WC-D15 as no major colour vision deterioration was detected by a preceding pseudoisochromatic plate test. It is to be further evaluated to what extent this is causally related to altitude exposure or the Waggoner software itself. However, the question whether the detection of colour vision changes beyond the sensitivity level of some validated colour vision tests still has practical relevance with respect to flight safety. This uncertainty can only be clarified by concrete investigations in the cockpit setting itself.

### Other influencing factors

Several observations such as the differences in susceptibility to develop AMS or high-altitude pulmonary oedema (HAPE) indicate that tolerance to hypoxia is an individual determent. A variety of different studies tried to reveal risk factor that predispose to vulnerability to hypoxic stress, amongst them, sex, age, SpO_2_, body mass index (BMI), anxiety or smoking can be found. Furthermore, several molecules were detected that might serve as potential biomarkers to predict high-altitude symptoms such as AMS including Insulin-like Growth Factor Binding Protein 6 (IGFBP6), Interleukin 12 Receptor A (IL-17RA), Hypoxia Inducible Factor 1 (HIF-1), Nitric Oxide (NO) or Heat Shock Protein 70 (HSP70) [50–52]. Whilst SpO_2_ can be considered as one of the few reliable influencing factors, data on the other parameters are controversial [53]. However, as stated before, no such investigations were yet performed specifically for colour vision. In the present study SpO_2_ values were recorded for each subject and at the various exposure altitudes. To date there are very little data on reference parameters for acute exposure to hypobaric hypoxia available nor are SpO_2_ values are given in most of the studies on colour vision in altitude. Camayo et al. published reference values for oxygen saturation from sea level to an altitude of up to 5100 m. Accordingly, a mean SpO_2_ level of 85% at 5000 m was found. In comparison a mean SpO_2_ level of 75% at 5000 m in the present study would be categorized below the 2.5th percentile in the former aggregate [54]. This is less surprising as Camayo et al. required a minimum of 2 months residence at the place of evaluation and numerous investigations have found SpO_2_ levels to normalize during the first days of exposure to altitude. Maximum standard deviation value in the present study was 4.07 and therefore comparable to figures in other altitude acclimatization studies [55]. The SpO_2_ levels within the exposure groups were not significantly correlated with the WCCVT and WC-D15 test results. Thus, individual tolerance to hypoxia did presumably not influence a subject’s test outcome. This in agreement with the before-mentioned study by Hovis et al. who could not detect any meaningful statistically significant relationship changes in the chromatic thresholds for the CCT or the CAD and age or SpO_2_ [45]. However, the results of a linear regression with such a small cohort size and partly insufficiently given statistical test prerequisites must be critically scrutinized.

### Limitations

There is an inter-observer variability of colour vision even amongst healthy subjects. Giving the relatively small sample size of 9–12 subjects per group, attribution of subtle changes to either altitude exposure or individual performance can be confounded. Mean age of the subjects was 27.3 whilst the age range was relatively large (mean ± SD 27.3 ± 7 years) but only 8 subjects were not between 20- and 30-year old. The aging process seems to have minimal effect on colour vision but an influencing impact on the present study cannot be ruled out with absolute certainty [56, 57]. In addition, all colour vision assessments require a minimum of attention and concentration ability. Negative influences of fatigue (especially in high altitudes) or elevated state of excitement could not be excluded [58]. At last, there is a certain lack of standardization in terms of display and colour selection as of different areas of confusion according to MacAdam ellipses. This applies even amongst manufacturers of “full glass cockpits” [5]. Thus, direct transfer of study results into practical applications is limited, especially when using a tablet computer of a type that is not commonly available in cockpits.

## Conclusions

Colour vision testing of 50 subjects using the Waggoner Computerized Color Vision Test (WCCVT) and the Waggoner D-15 (WC-D15) showed no significant deterioration in colour vision upon an exposure altitude of 15,000 ft over a maximum exposure duration of 60 min during a simulated flight in a decompression chamber. However, there was evidence of incipient impairment of colour vision above the aforementioned altitude. To clarify the nature of these tendencies, a larger number of participants will be necessary for similar investigations in the future. The results are inconsistent with a number of preliminary studies which partly showed significant deterioration starting from lower altitudes. However, it is important to note that the results of colour vision related altitude studies seem to be highly affected by the colour vision tests applied which show large differences in sensitivity and specificity, as well as susceptibility to user bias. In this attention the study scape is as manifold as the international regularities of the different national aviation authorities itself. Yet, it is important to account for the selected colour vision tests to investigate their strengths and weaknesses under (simulated) field conditions and eventually achieve a higher grade to standardization when it comes to pilot medical examinations. In this respect, the Waggoner test should also be subjected to further assessment in altitude environments. To provide a greater mechanistic insight into the underlying influencing factors that lead to colour vision deterioration in altitude, future experimental settings could be based on independent clinical measurements like SpO_2_. This might give the chance to factor out interobserver variability. On the other hand, such approaches would be confronted with a large number of possible (and not yet sufficiently confirmed) influencing variables to be considered.

As for the aeronautical context, detailed investigation into acute colour system adaptation must be conducted and its practical relevance to aviation safety critically evaluated. For this purpose, digital colour vision testing on a tablet PC holds the potential to make testing in pressure chambers or cockpits significantly more facilitated and economical and thus to generate larger amounts of data.

## Data Availability

The datasets used and analysed during the current study are available from the corresponding author on reasonable request.
